# Comparative Analysis of the MADS-Box Genes Revealed Their Potential Functions for Flower and Fruit Development in Longan (*Dimocarpus longan*)

**DOI:** 10.3389/fpls.2021.813798

**Published:** 2022-01-27

**Authors:** Baiyu Wang, Wenshun Hu, Yaxue Fang, Xiaoxi Feng, Jingping Fang, Tengyue Zou, Shaoquan Zheng, Ray Ming, Jisen Zhang

**Affiliations:** ^1^Center for Genomics and Biotechnology, Haixia Institute of Science and Technology, Fujian Provincial Key Laboratory of Haixia Applied Plant Systems Biology, College of Life Sciences, Fujian Agriculture and Forestry University, Fuzhou, China; ^2^Fujian Breeding Engineering Technology Research Center for Longan & Loquat, Fruit Research Institute, Fujian Academy of Agricultural Sciences, Fuzhou, China; ^3^College of Life Sciences, Fujian Normal University, Fuzhou, China; ^4^College of Mechanical and Electrical Engineering, Fujian Agriculture and Forestry University, Fuzhou, China; ^5^Department of Plant Biology, University of Illinois at Urbana-Champaign, Urbana, IL, United States

**Keywords:** longan, MADS-box, ABCDE model, KClO_3_, flower, fruit development

## Abstract

Longan (*Dimocarpus longan* Lour.) is an important economic crop widely planted in tropical and subtropical regions, and flower and fruit development play decisive effects on the longan yield and fruit quality formation. MCM1, AGAMOUS, DEFICIENS, Serum Response Factor (MADS)-box transcription factor family plays important roles for the flowering time, floral organ identity, and fruit development in plants. However, there is no systematic information of MADS-box family in longan. In this study, 114 MADS-box genes were identified from the longan genome, phylogenetic analysis divided them into type I (*M*α, *M*β, *M*γ) and type II (MIKC*, *MIKC^C^*) groups, and *MIKC^C^* genes were further clustered into 12 subfamilies. Comparative genomic analysis of 12 representative plant species revealed the conservation of type II in Sapindaceae and analysis of cis-elements revealed that *Dof* transcription factors might directly regulate the *MIKC^C^* genes. An ABCDE model was proposed for longan based on the phylogenetic analysis and expression patterns of MADS-box genes. Transcriptome analysis revealed that *MIKC^C^* genes showed wide expression spectrums, particularly in reproductive organs. From 35 days after KClO_3_ treatment, 11 *MIKC* genes were up-regulated, suggesting a crucial role in off-season flower induction, while *DlFLC*, *DlSOC1*, *DlSVP*, and *DlSVP-LIKE* may act as the inhibitors. The gene expression patterns of longan fruit development indicated that *DlSTK*, *DlSEP1/2*, and *DlMADS53* could be involved in fruit growth and ripening. This paper carried out the whole genome identification and analysis of the longan MADS-box family for the first time, which provides new insights for further understanding its function in flowers and fruit.

## Introduction

The MADS-box genes, widely distributed in fungi, plants, and animals, encode a large transcription factor family. These genes with diverse functions play important roles in plant development, signal transduction, and stress responses (Riechmann and Meyerowitz, 1997; Messenguy and Dubois, 2003). The family name “MADS” consists of the initials of its earliest genes, *MCM1* in yeast, *AGAMOUS* (*AG*) in *Arabidopsis thaliana* (Arabidopsis), *DEFICIEN*S in *Antirrhinum majus*, and *Serum Response Factor* (*SRF*) in humans (Passmore et al., 1988; Sommer et al., 1990; Yanofsky et al., 1990). Each MADS protein possesses a conserved domain of about 60 amino acids named MADS-box at the N-terminus, which recognizes and binds to CArG boxes (CC[A/T]6GG) (De Bodt et al., 2003). At an evolutionary level, all the members can be divided into two lineages, type I and type II, according to their relationships to animal *SRF-like* and *MEF2-like* genes, respectively (Alvarez-Buylla et al., 2000). In general, type I MADS genes have simple structures with 1–2 exons and there are few reports on their functions. The type II genes were also named *MIKC* genes due to their four domain structures: the highly conserved MADS-box (M) domain, the less-conserved intervening (I) domain, the moderately conserved keratin-like (K) domain, and the variable C-terminal (C) region (Theissen et al., 1996; Kaufmann et al., 2005). In Arabidopsis MADS-box genes were separated into five groups including *M*α, *M*β, *M*γ, *M*δ, and *MIKC* based on the phylogenetic analysis (Parenicova et al., 2003). These *MIKC* type transcription factors, only found in the plant, can be subdivided into *MIKC^C^* and MIKC* groups based on differences in I and K domains and have been characterized functionally in the regulation of growth and development (Theissen et al., 2000; Becker and Theissen, 2003). These type II genes may be separated from the ancestor of extant land plants (Becker and Theissen, 2003).

In seed plants, extensive research has been performed since the *MIKC^C^* type genes were demonstrated to be involved in floral organ identity, the control of flowering time, and seed development. They can be further classified into 12 clades (Becker and Theissen, 2003), for example, *SUPPRESSOR OF OVEREXPRESSION OF CONSTANS 1* (*SOC1*) and *SHORT VEGETATIVE PHASE* (*SVP*) participate in the regulation of flowering time; *APETALA 1* (*AP1*) is not only a floral meristem identity gene, but also the floral organ identity gene; *FRUITFULL* (*FUL*) regulates cell differentiation during fruit development (Coen and Meyerowitz, 1991; Gu et al., 1998; Lee and Lee, 2010). With extensive researches, the well-known ABCDE model was built from studies in Arabidopsis and *Antirrhinum majus*, which explains floral organ identity (Coen and Meyerowitz, 1991; Theissen, 2001; Theissen and Saedler, 2001). Within the floral meristem, A + E genes specify sepals, A + B + E genes specify petals, B + C + E genes determine stamens, and C + E genes direct carpels, in addition, D + E genes are involved in ovule development. In the model plant Arabidopsis, A-class genes are represented by *AP1*, *APETALA2* (*AP2*), *FUL*, *CAULIFLOWER* (*CAL*), and *AGAMOUS-LIKE 79* (*AGL79*) (Jofuku et al., 1994), B class correspond to genes from *APETALA3* (*AP3*) and *PISTILLATA* (*PI*) (Goto and Meyerowitz, 1994; Yang et al., 2003), C class includes *AG* (Yanofsky et al., 1990), D class includes *SHATTERPROOF* (*SHP*) and *SEEDSTICK* (*STK*) and E class include 4 *SEPALLATA* genes (*SEP1*, *SEP2*, *SEP3*, and *SEP4*) (Pelaz et al., 2000; Pinyopich et al., 2003; Ditta et al., 2004; Zahn et al., 2006).

Longan (*Dimocarpus longan* Lour., 2n = 2x = 30), a member of the Sapindaceae family, was derived and widely cultivated in Southeast Asia (Lai et al., 2000). As a famous subtropical fruit tree, longan commonly called “dragon eye” in China is of high nutritional and medicinal value (Mei et al., 2014). Floral induction (FI) is the biological basis for the development of floral organs and fruit. However, in longan, FI is sensitive to temperature and thus floral reversion occurs frequently (Chen et al., 2009). Began in the late 1990s, potassium chlorate (KClO_3_) has been heavily input into longan thanks to the discovery of flowering induction by KClO_3_ at almost any time of the year (Subhadrabandhu and Yapwattanaphun, 2001; Manochai et al., 2005). Interestingly, off-season FI by KClO_3_ was only found in longan, even its close species litchi (*Litchi chinensis* Sonn.) has never been affected by KClO_3_. Several studies have investigated the physiological mechanisms of FI by KClO_3_, indicating that starch, sucrose contents, and cytokinin may play an important role (Sringarm et al., 2009; Chang, 2010). In addition, “Sijimi” (“SJ”) longan, performs a unique trait of perpetual flowering (PF), flower and bears fruit throughout the year without external environment conditions (Zhang H.N. et al., 2016). Longan likely has a special FI mechanism among the perennial fruit species. In this decade, the molecular genetics of flowering in model plants has a great advanced development (Fornara et al., 2010), but the research progress of flowering regulation in perennial fruit trees is very limited due to their long juvenile period and complex genetic backgrounds. Several flowering genes have been cloned and analyzed in longans such as *FLOWERING LOCUS T* (*FT*), *AP1*, and *LEAFY* (*LFY*) (Winterhagen et al., 2013), however, no research has been carried out on the MADS-box family in longan.

Recently, we sequenced and assembled the “Shixia” (“SX”) longan genome, this chromosome level genome can assist us with the analysis of MADS-box genes from the entire genome. Here, we identified 114 MADS-box genes from the longan genome for the first time, with the phylogenetics, gene evolution, conserved motif, and cis-element analysis was performed. We also determined the expression profiles of longan MADS-box genes particularly the ABCDE model genes during off-season FI under KClO_3_ and fruit development. This work provides an overview and information useful for future functional analysis of longan MADS-box genes in the reproductive process.

## Materials and Methods

### Data Retrieval and Plant Materials

The Arabidopsis MADS-box genes were retrieved from The Arabidopsis Information Resource (TAIR)^1^. The MADS-box genes of *Amborella trichopoda*, *Citrus clementina*, *Citrus sinensis*, *Vitis vinifera*, *Oryza sativa*, *Sorghum bicolor*, *Zea mays*, *Musa acuminate*, *Nymphaea colorata*, and *Physcomitrella patens* were described in previous reports (Aono and Hasebe, 2006; Arora et al., 2007; Zhao et al., 2011; Hou et al., 2013; Project, 2013; Wang et al., 2015; Liu et al., 2017; Zhang et al., 2020). The genomic data of *Selaginella moellendorffii* and *Chlamydomonas reinhardtii* were obtained from Phytozome^2^ and *Xanthoceras sorbifolium* was downloaded from GigaDB^3^. The evolutionary relationships of the above species were obtained from National Center for Biotechnology Information (NCBI)^4^. The RNA-seq data from ten tissues (root, stem, leaf, dormant bud, flower bud, flower, young fruit, pulp, pericarp, and seed) of “Sijimi” (“SJ”) longan were downloaded from the NCBI under accession number: PRJNA329283 and PRJNA387674 from previous studies (Lin et al., 2017; Jue et al., 2019).

The collection of *Dimocarpus longan* and the performance of experimental research on such plants complied with the national guidelines of China. We collected apical buds under CK and KClO_3_ (99% active ingredient) treatment from 4∼6 years old “Shixia” longan in Maoming, Guangdong, China at ten-time points (from day 0 to day 54 including November 18, 2016, November 23, 2016, November 28, 2016; December 3, 2016, December 8, 2016, December 13, 2016, December18, 2016, December 23, 2016, December 29, 2016, and January 11, 2017). Four whorls of floral organs including sepal, petal, stamen, carpel (pistil without ovary) and ovary of “Baoshi No. 1” (“BS-1”) and fruit of n were collected in Germplasm Repository of Longan (*Dimocarpus longan*), Fuzhou City, Ministry of Agriculture. All samples were collected and stored at −80°C until RNA-Seq. A pair-end library was made using the Illumina^®^ TruSeq™ RNA Sample Preparation Kit [RS-122-2001(2), Illumina] and sequencing using Illumina X ten.

### Identification and Features of MADS-Box Family in Longan

Two strategies were used to identify the MADS-box transcription factor family: the hidden Markov model (HMM) profile of SRF-TF domains (PF00319) from the Pfam database^5^ was used as a query to identify MADS-box sequences with HMMER version 3 (Eddy, 2011) against the longan genome with a threshold of e-value ≤ 1 × 10^–5^. In addition, MADS protein sequences of Arabidopsis were used as queries to search against the longan genome using the BLASTP program (Altschul et al., 1990) with an e-value cutoff of 1e−5 and identity > 40%. Subsequently, these proteins were submitted to the Pfam database and NCBI Conserved Domain Search^6^ to confirm the presence and completeness of the MADS domain. Candidate genes without the MADS domain were re-annotated manually with the assistance of FGENESH (Solovyev et al., 2006). The physical-chemical properties of longan MADS-box genes were predicted with the online tool ProtParam from ExPASy (see text footnote 6).

### Phylogenetic Analysis

Sequence alignments of MADS-box genes were performed using the MUSCLE program in MEGA X (Kumar et al., 2018) with default parameters. The NJ (neighbor-joining) phylogenetic tree was constructed using MEGA X with the following parameters: Poisson model and pairwise deletion, bootstrap for 1000 replicates. Maximum-likelihood (ML) phylogenetic trees were constructed using FastTree software (Price et al., 2009) with the LG model. Arabidopsis MADS-box genes *(AtMADS)* were used to assist classification (Theissen et al., 2000).

### Chromosome Locations and Synteny Analysis

To analyze the synteny for longan MADS-box genes (*DlMADS*), the BLASTP program (e-value < 1e−5), and MCScanX (Wang et al., 2012) were used. The tool ‘duplicate gene classifier’ was used to classify the origins of duplicate genes for longan.

### Gene Structure, Conserved Motif, and Cis-Element Analysis

Gene structures of MADS-box genes were extracted from the General Feature Format (GFF) file and the diagram was drawn with the online program Gene Structure Display Server (GSDS^7^). Conserved motifs were identified using Multiple EM for Motif Elicitation (MEME, version 5.3^8^) with the following parameters: 10 different motifs, Minimum Motif Width 10, Maximum Motif Width 100. The identified motifs were sent to Interpro^9^ for annotation.

The 1,500-bp sequences upstream of the start codon of each longan MADS-box gene were extracted to predict cis-acting elements through PlantCARE^10^. We further detected the conserved motifs from promoters of *MIKC^C^* genes using MEME with *p*-values < 0.05 and compared the motifs with known transcription factor binding sites (TFBS) from JASPAR Core plants 2018 database^11^ by performing Motif Comparison Tool TOMTOM (TOMTOM version 5.3^12^) with e-value < 0.05.

### RNA-Seq Data Analysis

Raw reads from RNA-seq were trimmed with Trimmomatic version 0.39 (Bolger et al., 2014) to remove adaptor sequences and low-quality reads. The high-quality reads were mapped to the “SX” longan genome using HISAT2 version 2.1 (Kim et al., 2015). The expression levels of MADS-box genes were normalized to fragments per kilobase of exons per million fragments mapped (FPKM) using StringTie version 2.1.2 (Kovaka et al., 2019). We defined genes with an FPKM value < 1 as not expressed, genes with FPKM value > 1 as lowly expressed, and values > 10 as highly expressed. Genes with FPKM value > 100 were extremely highly expressed. The differentially expressed genes (DEGs) were identified using the R package DESeq2 version 1.3 (Love et al., 2014). The gene co-expression networks were constructed using the WGCNA (Langfelder and Horvath, 2008) package with the filtered genes (FPKM > 1 at least one sample). Software Cytoscape version 3.7.1 (Shannon et al., 2003) was used to visualize the gene interaction networks of MADS-box genes.

### Expression Validation of *DlMADS* by qRT-PCR

The cDNA for qRT-PCR was synthesized using the StarScript II First-strand cDNA Synthesis Mix with gDNA Remover (GenStar, A224-10). Gene-specific primers were designed using the online tool PrimerQuest^13^ (Supplementary Table 1). Two longan actin genes (*Dil.10g021740.1* and *Dil.06g016430.1*) were used as the internal control and three replicates were performed (Jue et al., 2019). The qRT-PCR amplification was carried out using 2× RealStar Green Fast Mixture (GenStar, A301-10) on a Multicolor Real-Time PCR Detection System (Bio-Rad) using the protocol for this kit: 95°C for 2 min, 40 cycles of 95°C for 15 s and 60°C for 30 s. The relative expression levels of the candidate genes were calculated using the 2−ΔΔCt method.

## Results

### Identification of MADS-Box Genes in Longan

To identify the MADS-box gene family, both the hidden Markov model (HMM) profile (PF00319) and 107 Arabidopsis MADS-box protein sequences were used as queries to perform HMMER and BLASTP against the “SX” longan genome. A total of 117 candidate MADS-box genes were identified. Among them, nine genes that did not have MADS domain-coding sequences were manually re-annotated with the assistance of the online tool FGENESH. Three re-annotated sequences without MADS domain were excluded from further analysis. Finally, 114 complete MADS-box genes were confirmed and named as *DlMADS1-DlMADS114* based on their genomic location in the chromosome (Supplementary Table 2). The length and molecular weight of 114 MADS-box proteins ranged from 64 AA and 7358.57 Da (*DlMADS85*) to 643 AA and 72378.92 Da (*DlMADS101*), with isoelectric points in the range of 5.06 to 10.49 (Supplementary Table 2). This result showed divergences in physicochemical properties among MADS-box family members in longan.

### Phylogenetic and Evolutionary Analysis

To classify and examine the evolutionary relationship among MADS-box genes, we constructed a neighbor-joining (NJ) phylogenetic tree with alignments of longan and Arabidopsis MADS-box protein sequences. The result showed that *DlMADS* were divided into 5 groups, as in Arabidopsis (Parenicova et al., 2003; Supplementary Figure 1). Of these, 63 longan MADS-box genes were assigned to type I including 39 genes in *M*α, 10 genes in *M*β, 14 genes in *M*γ. 51 *DlMADS* were classified as type II including 36 *MIKC^C^* and 15 MIKC* type genes.

**FIGURE 1:**
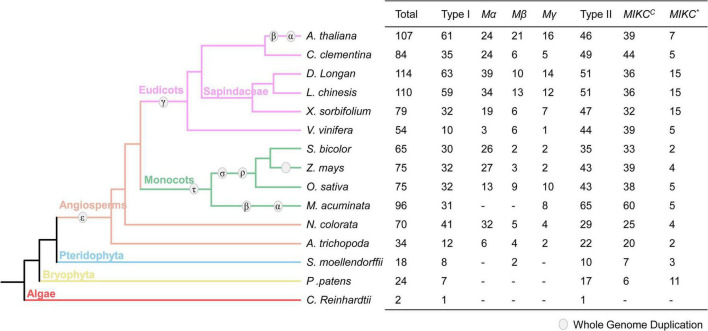
The MADS-box gene family in 15 species. The evolutionary relationship of 15 species on the left of the figure was obtained from NCBI (https://www.ncbi.nlm.nih.gov/Taxonomy/CommonTree/wwwcmt.cgi). The right of the figure shows the number detail of the MADS-box family in each species. Type I MADS-box genes contain three groups: *M*α, *M*β, *M*γ, and type II genes are subdivided into *MIKC^C^* and MIKC* groups.

The MADS-box family is widely distributed in plants, and to understand the gene evolution in longan, we searched for MADS-box genes in the genomes of 15 representative plant species for comparative genomic analysis. Nine of them have been previously reported (see Methods), while MADS-box genes of the other six plant species were identified and classified from their genomes. As shown in Figure 1, both type I and type II have single genes in *C. reinhardtii* (Algae), supporting the view that these two types of MADS-box genes are very ancient and originated before the origin of Embryophyte. The MADS-box genes were expanded in Angiospermae due to the ε-whole-genome duplication (WGD). Compared to type I, the number of type II genes is relatively conserved in monocots and eudicots. In addition, longan has the largest number (114) of MADS-box genes, similar to litchi.

**FIGURE 2:**
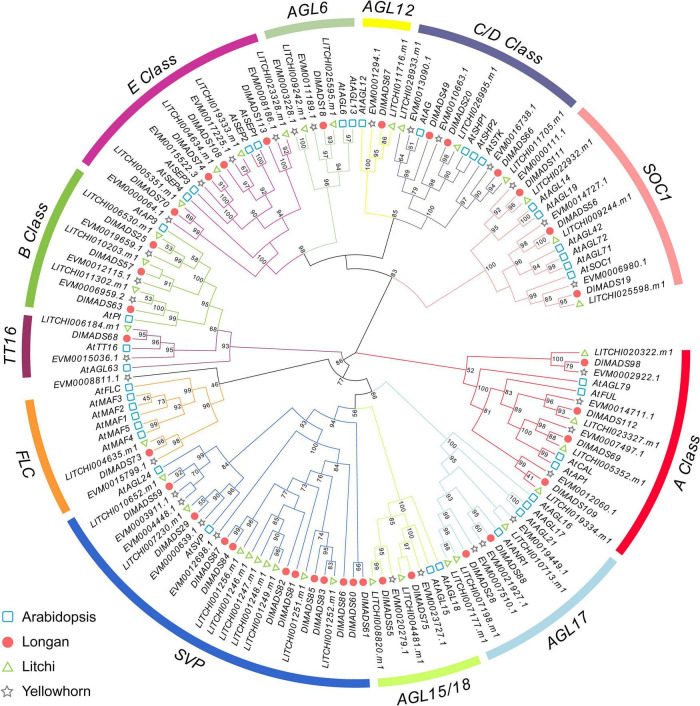
Phylogenetic analysis of type II MADS-box transcription factors. The maximum-likelihood (ML) tree was constructed with *MIKC^C^* proteins sequences from Arabidopsis (*At*), longan (*Dl*), litchi (*LITCHI*), and yellowhorn (*EVM*).

In order to further classify the *MIKC^C^* genes, two phylogenetic trees were constructed using MADS-box proteins from Arabidopsis, longan, litchi, and *Xanthoceras sorbifolium* (yellowhorn) using the maximum-likelihood (ML) and neighbor-joining (NJ) methods (Figure 2 and Supplementary Figure 2). The structures of the two trees based on NJ and ML methods were similar, indicating a reliable subfamily division. The longan *MIKC^C^* genes could be divided into 12 subgroups with Arabidopsis genes as a reference, and no novel subfamily was found, indicating the conservation of the gene evolution in dicot species. The *Flowering Locus C* (*FLC*) clade of Arabidopsis contains one *FLC* gene and five homologs, *MADS-AFFECTING FLOWERING 1-5* (*MAF1-5*), which was attributed to the two rounds of Arabidopsis specific WGD. Only one gene was found in each Sapindaceae *FLC* clade, which was similar to the result in Citrus (Hou et al., 2013). Similar to *FLC*, subgroups *TT16* and *AGAMOUS-like 6* (*AGL6*) contained the minimum number (one) of longan type II genes. For the *SVP* clade, there are two genes (*SVP* and *AGL24*) in Arabidopsis, with the *SVP* subgroup consisting of the largest number (11) of *MIKC^C^* type genes in longan, indicating that some of the loss and duplication events probably occurred after the divergence of two the species. As *dormancy-associated MADS-box* (*DAM*) genes, which were highly homologous to *SVP* and *AGL24*, have been proven to affect dormancy in peach (Bielenberg et al., 2008), we collected reported *DAM* gene sequence data from *Prunus persica* (peach), *Prunus mume* (plum), *Pyrus pyrifolia* (pear), and *Malus domestica* (apple) to generate a phylogenetic tree with longan and litchi (Supplementary Figure 3). Except for the main *SVP* and *AGL24* clade, all the Rosaceae *DAM* genes cluster in one clade, with nine longan MADS genes (named as *DlSVP-LIKE*) and seven litchi MADS genes located in an individual cluster.

### Genomic Distribution and Gene Duplication

**FIGURE 3:**
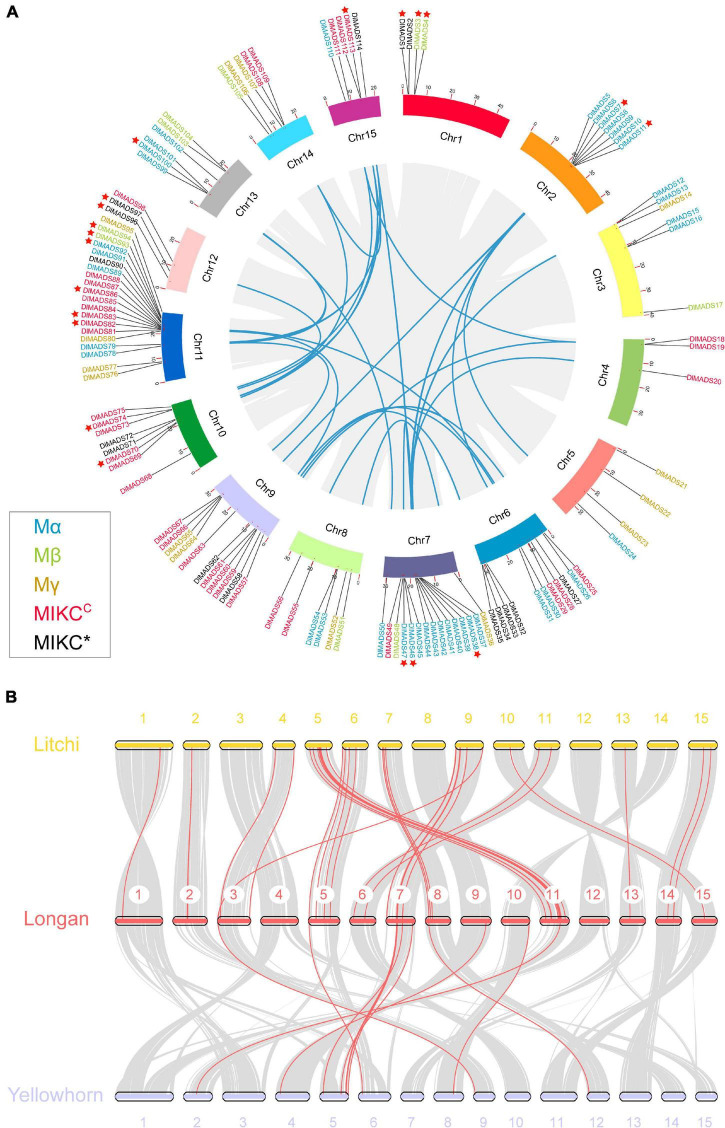
Chromosomal location and synteny analysis of the MADS-box genes. **(A)** A total of 114 MADS-box genes are located in 15 chromosomes with different colors. Gene pairs of WGD or segmental duplication are linked using blue lines. Tandem duplication genes are marked by red stars. Gene labels of five types: *M*α, *M*β, *M*γ, MIKC*, and *MIKC^C^* are denoted by blue, green, yellow, red, and black, respectively. **(B)** Synteny relationship of type I MADS-box genes from longan, litchi, and yellowhorn genomes. Gray lines in the background indicate the collinear blocks within two genomes. Red lines highlight the syntenic type I MADS-box gene pairs.

The 114 MADS-box genes were unevenly distributed on 15 chromosomes of the longan genome (Figure 3A). At least 3 *DlMADS* were found in each chromosome, chromosome 11 had the largest number of family members at 20, and only three genes were located on chromosomes 4 and 12. Gene duplication has been considered the driving force for species evolution, and WGD events have probably occurred in many eukaryotes, sometimes more than once (Van de Peer, 2004). Previous research has established that over 90% of the increase in the number of regulatory genes was caused by the three WGD events in Arabidopsis (Maere et al., 2005). In this study, we investigated gene duplication events in Arabidopsis, longan, litchi, and yellowhorn genome using MCScan X. Of the genes of the longan MADS-box family, 44 (38.6%) originated from WGD or segmental duplication, 20 (17.5%) appeared to have been created through tandem duplication, 22 (19.3%) were proximal duplicated genes and 28 (24.6%) were dispersed duplicated genes (Table 1). In Arabidopsis, nearly half of the MADS-box genes (46.7%) originated from dispersed duplication, while tandem duplication events were more widespread in the MADS-box genes which indicates they made a valuable contribution to the evolution of the longan MADS-box family. Of significance, at least 26 genes of the *M*α group were clustered on special regions of chromosomes 2, 7, and 13, indicating that tandem duplications were the main force driving the expansion of this MADS-box group.

**TABLE 1:** Numbers of MADS-box genes from different origins in Arabidopsis, longan, litchi, and yellowhorn genomes.

	Number of MADS-box genes	Number of genes from different origins (percentage)				
		Singletons	WGD/Segmental	Tandem	Proximal	Dispersed
*Arabidopsis thaliana*	107	4 (3.7%)	24 (22.4%)	11 (10.3%)	18 (16.8%)	50 (46.7%)
*Dimocarpus longan*	114	0 (0.0%)	44 (38.6%)	20 (17.5%)	22 (19.3%)	28 (24.6%)
*Litchi chinensis*	114	1 (0.1%)	18 (15.8%)	27 (23.7%)	14 (12.3%)	54 (47.4%)
*Xanthoceras sorbifolium*	79	1 (0.1%)	22 (27.8%)	13 (16.5%)	8 (10.1%)	35 (44.3%)

In Sapindaceae, type II gene numbers were generally conserved, while type I gene numbers varied. Longan and litchi have consistent numbers of MADS-box genes in all the subgroups with the exception of *M*β and *M*γ (Figure 1). Yellowhorn had much fewer type I MADS-box genes (32) than both longan (63) and litchi (59). Therefore, we constructed the comparative syntenic maps among longan, litchi, and yellowhorn (Figure 3B). For type I, 44 orthologous gene pairs were identified between longan and litchi, indicating close relationships. In comparison to longan and litchi genomes, gene concentration was observed in which was mainly caused by the loss of type I genes in the corresponding longan chromosomes 1, 2, 3, 6, 11, 13, 14, and 15. Moreover, the synonymous substitution rates (Ka/Ks) of the gene pairs were calculated to identify the evolutionary forces. All of the 58 orthologous gene pairs had Ka/Ks < 1 (Supplementary Table 3), suggesting that purifying selection may be the dominant force driving the evolution of Sapindaceae type I MADS-box genes.

### Gene Structure and Conserved Motifs of Longan MADS-Box Family

To assess the structural diversity of longan MADS-box genes, the GSDS program was used to display intron-exon organization. The results showed that the structures of type I genes were short and simple with the presence of 0–2 introns except for *DlMASD101* with 9 introns (Supplementary Figure 4). The full-genome lengths of *DlMADS40* and *DlMADS42* were significantly longer at 6503 and 9040 bp. Through the survey of transposable element (TE) sequences, we found that 9 and 5 TEs were located in *DlMADS40* and *DlMADS42*, respectively (Supplementary Table 4). Compared with type I, type II genes contain more exons in the range of 0–12, which is in contrast to the previous report that *MIKC* genes have a common structure of 1–6 exons (Johansen et al., 2002). In addition, the average length of MIKC* genes (3,900 bp) was shorter than *MIKC^C^* genes (8,800 bp).

**FIGURE 4:**
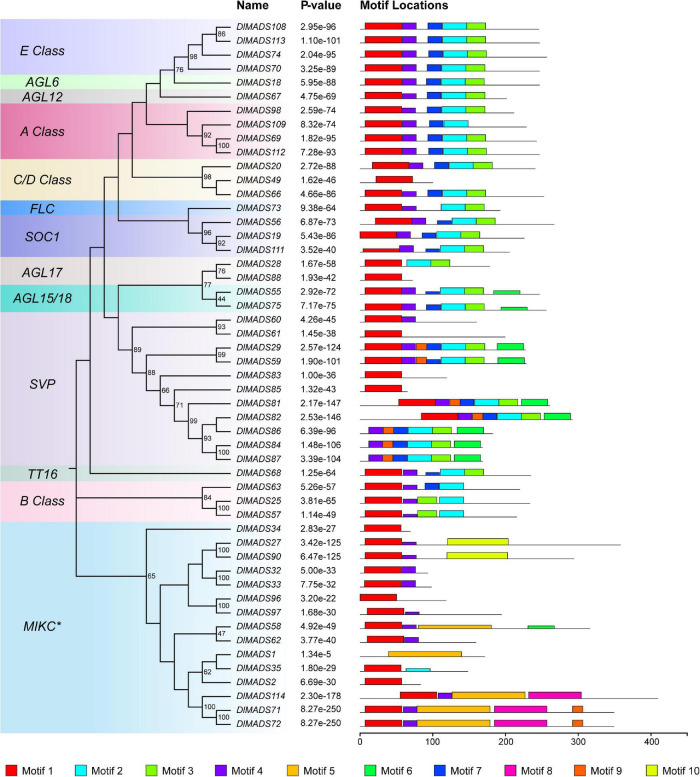
Conserved motif compositions of longan type II MADS-box proteins. The neighbor-joining tree was constructed with the aligned protein sequences of longan type II MADS-box genes. 10 motifs were identified and displayed in different colors.

Since *MIKC* type genes with complex structures play functional roles in developmental processes in plants (Becker and Theissen, 2003), we analyzed the conserved motifs of longan *MIKC* type proteins using the MEME program. A total of 10 conserved motifs were identified among 51 *MIKC* protein sequences (Figure 4 and Supplementary Table 5) and the number of motifs in *DlMADS* ranged from 1 to 7 and each subfamily had similar motif compositions. Motif 1 which was annotated as MADS domain was found in nearly all protein sequences except for *DlMADS86*, *84*, *87* in the *SVP* class and *DlMADS1* in the MIKC* class, with the distribution of the unknown motif 4 being similar to motif 1, suggesting that these two motifs play crucial functions. Motif 2, 3, and 7 may be fragments of the K-box and are distributed in most *MIKC^C^* members, but motif 5 annotated as K-box was only found in MIKC* class. The result reveals that the primary differences between *MIKC^C^* and MIKC* proteins are variations in the K domain.

### Cis-Elements and Potential Transcription Factor Binding Sites

**FIGURE 5:**
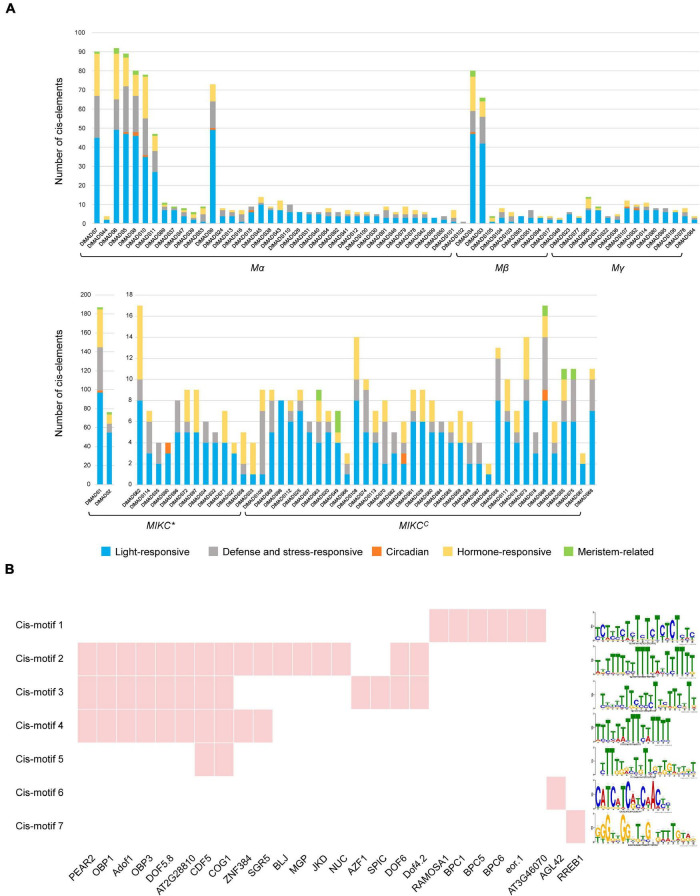
Prediction of cis-elements and transcription factors of *DlMADS*. **(A)** The number of cis-elements related to light-responsive, defense and stress-responsive, circadian, hormone-responses and meristem. The upstream 1.5 kb sequences of all longan MADS-box genes were analyzed through PlantCARE. **(B)** The cis-motifs and transcription factors potentially target 36 *MIKC^C^* genes. Red indicates the transcription factor binding sites were found in promoter motifs of *MIKC^C^* genes.

Transcription factors (TFs) control and regulate gene expression through binding cis-elements in the promoters of target genes. To investigate the regulatory gene networks of the MADS-box family, we analyzed the cis-elements of the upstream 1,500-bp sequences of *DlMADS* based on the PlantCARE database. Cis-elements associated with 11 biological processes such as light-responsiveness and the circadian clock were annotated in *DlMADS* (Supplementary Table 6). Of note, the number of functional elements in *MIKC^C^* type genes is significantly less than that of other genes (Figure 5A). Light-responsive boxes existed in all MADS-box genes. Hormone-related (ABRE, TGA-element, AuxRR-core, GARE-motif, P-box, etc.) and defense and stress-responsive (WUN-motif, LTR, TC-rich repeats, ARE) cis-elements were found in most family members. Additionally, *DlMADS1* belonging to the MIKC* group had the most cis-elements amongst the gene families. In the *MIKC^C^* subfamily, the hormone-related elements were more frequently located in *SEP*, *FLC*, and *SVP* clades.

Transcription factors can directly bind to specific sequences in promoters of target genes called binding sites to affect gene expression. In order to further identify the transcription factors which potentially control the *MIKC^C^* type genes, the promoters of 36 *MIKC^C^* genes were processed using MEME software to locate cis-motifs. As a result, 20 enriched motifs were found and a total of 26 TFs along with their DNA bind sites were identified in 7 motifs by comparison with the JASPAR database (Figure 5B and Supplementary Table 7). 21 TFs were *C2H2* zinc finger proteins particularly the DNA-binding *One Zinc Finger* (*Dof*) transcription factor family. For example, *CYCLING DOF FACTOR 5* (*CDF5*) and *COGWHEEL1* (*COG1*) binding sites were found in 4 motifs. Other TFs belong to *BARLEY B RECOMBINANT/BASIC PENTACYSTEINE* (*BBR/BPC*), MADS-box family and, ETS-related genes.

### A Proposed ABC(D)E Model in Longan

**FIGURE 6:**
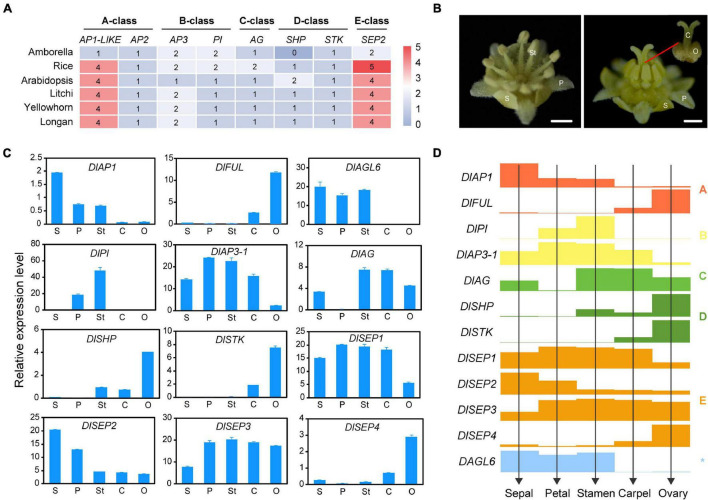
The deduced ABCDE model in longan. **(A)** The heat map shows the number of ABCDE classes genes in Amborella, Arabidopsis, rice, and longan. **(B)** Mature male (left) and female (right) flowers of longan. Five types of floral organs including sepal (S), petal (P), stamen (St), carpel (C), ovary (O) were collected for gene expression analysis. **(C)** Expression analysis of ABCDE classes genes in floral organs in **(B)** by using qRT-PCR. **(D)** The ABCDE model in longan is proposed based on the gene expression levels (bar heights) from **(C)**.

Extensive research showed that five classes of homeotic genes called the ABCDE model, control floral development at the molecular level. Phylogenetics and classification based on the model plant as reference allowed the identification of the putative functions of longan MADS-box genes. As shown in Figure 6A, only one copy of an A-class functional MADS-box gene was found in Amborella and four homologs were confirmed in rice, Arabidopsis, and the three Sapindaceae species. Phylogeny indicated that A-class genes were present in a one-to-one orthologous pattern in the Sapindaceae species. *AP1* and *CAL* originated from a recent duplication event and have both partially redundant and unique functions (Alvarez-Buylla et al., 2000), and only one gene of longan (*DlMADS69*) was found in *AP1/CAL* clade, named as *DlAP1*. In addition, the number of *AP2* genes in the *AP2/EREBP* family was highly conserved in tested Angiosperm (Figure 6A). Both *AP3* and *PI* which diverged in the ancestors of angiosperms experienced loss events in eudicots, and C and D class genes formed a very close sister group, with each Sapindaceae species having one *AG*, one *STK*, and one *SHP* gene. *SEP* genes have roles throughout five whorls of floral organs and were duplicated during angiosperm evolution. Four *SEP* homologs were found in longan, litchi, and yellowhorn. Overall, these results demonstrated that the number of ABCDE genes in Sapindaceae was similar to the model plant Arabidopsis.

In order to investigate the correlation between ABCDE genes and longan floral organs, we analyzed the expression patterns of certain genes in mature four whorls of floral organs (sepals, petals, stamens, carpel) and ovary by performing qRT-PCR (Figures 6B,C). Although twelve selected genes could be detected in most organs, they showed high specificity in different organs. For A-class, *DlAP1* was mainly expressed in the first three whorls and *DlMADS112* (*DlFUL)* was mainly expressed in carpel and ovary. *DlMADS63* (*DlPI*) of B class was only detected in petal and stamen, *DlMADS25* (*DlAP3-1*) also showed relatively high expression in these two organs, even though it could be found in all five organs. The only C class gene *DlMADS49* (*DlAG*) was detected in sepal, stamen, carpel, and ovary, especially in stamen and carpel. Two D-class genes, *DlMADS20* (*DlSHP*) and *DlMADS66* (*DlSTK*) were expressed in reproductive organs (stamen and pistil) and showed relatively high expression levels in the ovary which contains ovules. Interestingly, four members of the E class showed different expression patterns in floral organs suggestive of diversified gene function. The expression levels of *DlMADS74* and *113* (*DlSEP1* and *3*) were well-proportioned in five orangs. However, *DlMADS108* (*DlSEP2*) showed a similar expression pattern with *DlAP1*, and *DlMADS70* (*DlSEP4*) was preferentially expressed in the ovary like *DlSTK*.

Besides the ABCDE genes, *AGL6* has been demonstrated to control floral development. In Arabidopsis flowers, *AGL6* could be detected in all floral organs and in developing ovules (Koo et al., 2010). However, the transcripts of *DlMADS18* (*DlAGL6)* were only detected in the first three whorls of organs (sepals, petals, and stamens), which suggested that this gene may not control longan ovule development. These results allowed us to predict the ABC(D)E model in longan (Figure 6D).

### Tissue-Specific Expression Patterns of *DlMADS*

To investigate the expression pattern of longan MASD-box genes, previously published transcriptome data from multiple tissues including root, stem, leaf, dormant bud (before the emergence of floral primordia), flower bud, flower, young fruit, pericarp, pulp, and the seed of “SJ” longan were used for analysis (Lin et al., 2017; Jue et al., 2019). In line with previous studies (Kofuji et al., 2003; Duan et al., 2015), almost all genes from type I displayed low expression levels (FPKM value < 1) in nine tissues (Supplementary Table 8). In addition, *DlMADS21* was highly expressed specifically in roots and *DlMADS53* showed extremely high expression in pulp (Supplementary Figure 5), which suggests that these two genes play roles in root and fruit development, respectively. Moreover, *DlMADS45* was primarily expressed in root, stem, and seeds. *DlMADS65* was lowly expressed in multiple tissues. In the MIKC* group, *DlMADS71*, *72*, *114* with more protein motifs in the K-box region than others showed higher expression in nearly all tissues, which provides evidence to the fundamental role of the K-box domain. The expression level of *DlMADS27* and *DlMADS58* in flower buds and flowers were higher than other organs, indicating crucial roles in the flowering process.

**FIGURE 7:**
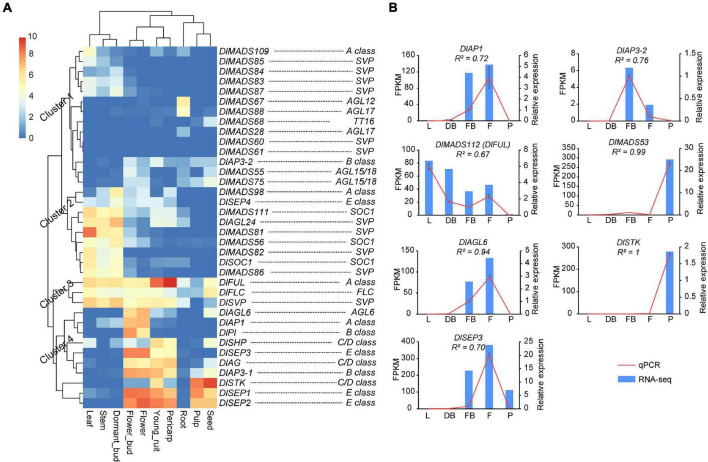
The expressional pattern of *MIKC^C^* genes in longan. **(A)** Expression heat maps of *MIKC^C^* genes in nine tissues (root, stem, leaf, dormant bud, flower bud, flower, fruit, pericarp, and seed) of “SJ” longan Expression values were normalized by log_2_(FPKM+1). **(B)** Detection of MADS-box genes in longan based on qRT-PCR. Six MADS-box genes were selected to detect the expression level in leaf (L), dormant bud (DB), floral bud (FB), flower (F), and pulp (F) of “XC” longan. Data were normalized to Actin1 and Actin2 genes, and the vertical bars indicate standard deviation.

*MIKC^C^* type genes have important functions in the productive organs in plants (Becker and Theissen, 2003). In longan, genes of *MIKC^C^* displayed a wide range of expression levels in different tissues, among which 11 (30.6%), 18 (50.0%), 17 (47.2%), 17 (47.2%), 24 (66.7%), 20 (55.6%), 16 (44.5%), 16 (44.5%), 8 (22.2%), 14 (38.9%), and 16 (44.5%) were expressed (FPKM value > 1) in root, stem, leaf, dormant bud, flower bud, flower, young fruit, pulp, pericarp and seed, respectively (Supplementary Figure 6). Although expression patterns of *DlMADS* were conserved within each subfamily, several genes showed different expression levels in tested tissues such as *DlAP1*, *DlFUL*, and *DlMADS109* in the A-class group. Our analysis showed that expression patterns of *MIKC^C^* genes in longan tissues were classified into four clusters (Figure 7A). In cluster 1, most genes show little expression but several genes such as *SVP* class members were expressed in leaf, stem, and dormant bud. *DlMADS67* and *DlMADS88* were specifically expressed in roots. In cluster 2, *DlMADS* was mainly expressed in vegetative tissues. *DlMADS73* (*DlFLC*), *DlFUL*, and *DlMADS29* (*DlSVP*) of cluster 3 displayed extensive-expression levels in nearly all tested tissues. In cluster 4, *DlAGL6* and 9 genes of the ABCDE classes were mainly expressed in the reproductive organs including flower bud, flower, fruit, pericarp, and seed. From this finding, it is clear that *MIKC^C^* type *DlMADS* might perform significant roles in flowering and fruit development as opposed to other organs.

To further survey the expression patterns of key *DlMADS* having reliable transcriptional support, quantitative RT-PCR was performed using total RNA isolated from leaf, dormant bud (before the emergence of floral primordia), floral bud (floral primordia), flower, and pulp (Figure 7B). Among seven selected genes, *DlAP1*, *DlMADS57* (*DlAP3-2*), and *DlAGL6* were only detected in floral or flower tissues, suggesting a unique function in flowering. *DlSEP3* showed high expression in the floral bud, flower, and pulp tissues. Moreover, *DlMADS53* showed specific high expression in pulp, consistent with the result of RNA-seq.

### Differential Expression of *DlMADS* in Off-Season Flower Induction

The compound potassium chlorate (KClO_3_) has been widely used to induce flowering in longan since the last century (Subhadrabandhu and Yapwattanaphun, 2001). In this study, we detected the expression of MADS-box genes in apical buds at ten-time points using untreated controls (CK) and KClO_3_ treatments (see Methods). A total of 34 genes were expressed in more than one stage (FPKM > 1), including 26 *MIKC^C^* type genes, 3 MIKC* genes, and 5 genes of type I (Supplementary Table 9).

**FIGURE 8:**
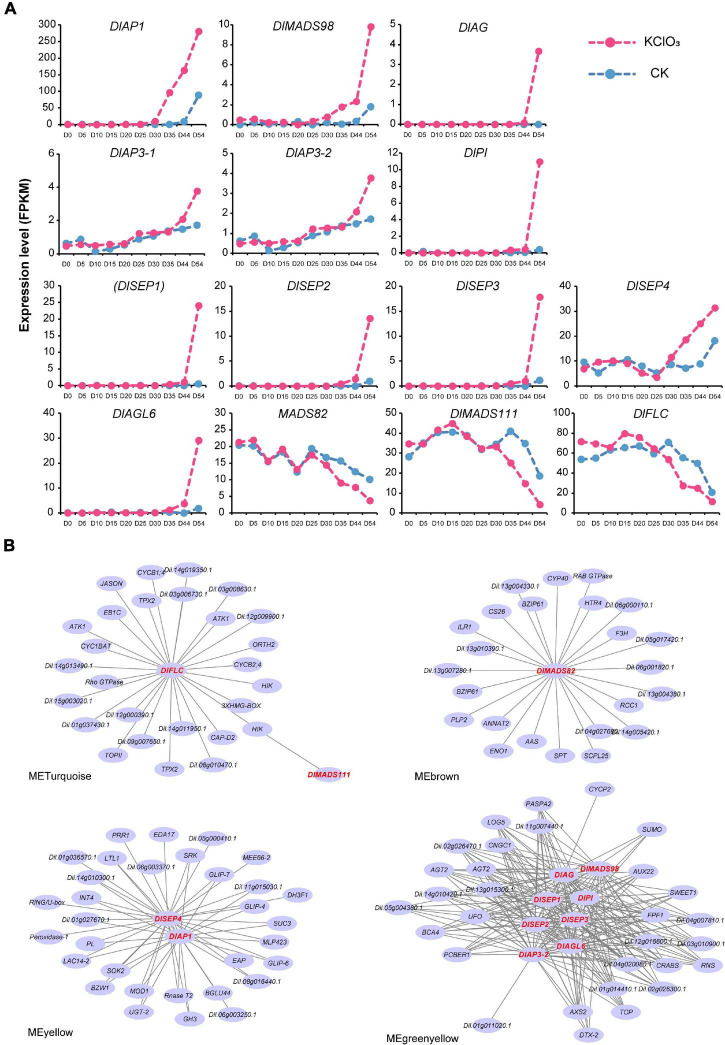
Transcript analysis of longan MADS-box genes in off-season flower induction. **(A)** Expression profile of differentially expressed MADS-box genes during 10 stages (day 0∼54) under CK and KClO_3_ treatment. **(B)** Co-expression networks of differential expression MADS-box genes during off-season flower induction. Each node represents a gene and the lines between nodes represent co-expression correlations.

Among these, we found 14 genes showed significant differential expression levels (| logFC| > 2 and *P*-value < 0.05) after KClO_3_ treatment, including the ABCE model genes and other flowering time integrators (Figure 8A). *DlAP1*, *DlMADS98*, and *DlSEP4* were up-regulated from day 41 to day 54, especially *DlAP1* with the highest FPKM value. It is interesting to note that *DlAP3-1*, *DlAP3-2*, *DlPI*, *DlAG*, *DlSEP1/2/3*, and *DlAGL6* showed the same patterns with upregulation on day 54. Therefore, these up-regulated genes could be considered as promoting factors for off-season flowering induction. In addition, *DlMADS111*, *DlFLC*, and *DlMADS82* displayed a decreased pattern from day 35 to day 54, suggesting that these may inhibit longan flowering in this time period.

To assess the relationships between *DlMADS* and other genes throughout the genome that may affect off-season flowering, the expressed genes were classified into 16 modules by weighted gene co-expression network analysis (WGCNA) (Supplementary Figure 7). The result showed that 14 differentially expressed MADS-box genes were distributed in four modules (MEyellow, MEbrown, MEturquoise, and MEgreenyellow). Based on the differentially expressed MADS-box genes, we constructed the co-expression networks and found that 113 genes were presumed to interact with them (Figure 8B). Based on the *Arabidopsis thaliana* flowering interactive database^14^, we found five flower-related genes interacted with MADS-box genes in the networks including *UNUSUAL FLORAL ORGANS* (*UFO*), *TEOSINTE BRANCHED1/CYCLOIDEA/PROLIFERATING CELL FACTOR* (*TCP*), *CRABS*, *FLOWERING PROMOTING FACTOR1* (*FPF1*), and *HOTHEAD* (*HTH*) (Kania et al., 1997; Krolikowski et al., 2003; Hepworth et al., 2006; Gross et al., 2018; Zhao et al., 2020). Besides these flower-related genes, (*UDP-Glycosyltransferase*-2) *UGT-2*, (*SPATULA*) *SPT*, and *SWEET1* were predicted to co-expressed with the MADS-box genes although have not yet been reported for the function related to flower. Whether these genes play functions in longan flower induction and development needs more experiments to confirm. In addition, 8 *DlMADS* (*DlMADS98*, *DlAG*, *DlSEP1/2/3*, *DlPI*, *DlAP3-2*, and *DlAGL6*) were found in the green-yellow module and *DlAGL6* was the top hub gene with the highest kME value (0.99) indicating the central role in flower development (Figure 8B).

Moreover, we also compared the expression levels of *DlMADS* between “SX” and “SJ” during three floral transition stages (T1: dormant bud; T2: floral primordia; T3: floral organ formation). As a result, seven MADS-box genes were significantly differentially expressed in two accessions (Supplementary Figure 8). The expression of *DlMADS111, DlAGL24, DlFLC*, and *DlSOC1* was higher in “SX_T1” than that in “SJ_T1,” and they were all downregulated in the following two stages except for *DlSOC1*. *DlAP1* was upregulated in T1 and T2 in both accessions but showed higher expression levels in “SX” than that in “SJ.” On the contrary, the *DlMADS98* displayed a higher expression in “SJ” than that in “SX” in the entire stages.

### Expression Profiling of *DlMADS* During Fruit Development

**FIGURE 9:**
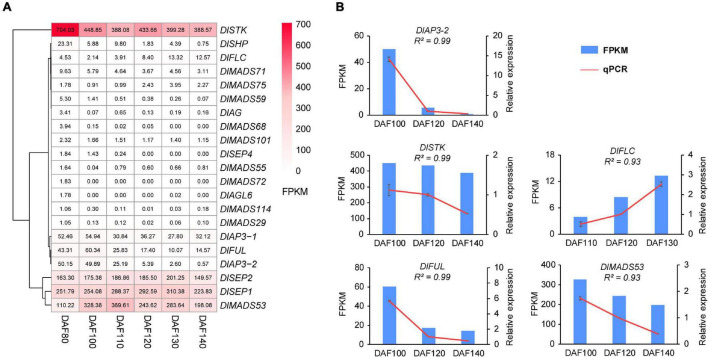
The expression pattern of differentially expressed *DlMADS* during the fruit development stage. **(A)** Heat maps based on FPKM for differentially expressed *DlMADS* at three fruit developmental stages (80∼140 days after flowering). **(B)** Detection of five differentially expressed *DlMADS* at three selected stages by using qRT-PCR. The bar and line graphs are derived from RNA-seq and qRT-PCR data, respectively.

It was reported that the MADS-box gene family have functions in fruit development and ripening (Moore et al., 2002), and our present study analyzed the expression pattern of *DlMADS* in six fruit developmental stages at 80, 100, 110, 120, 130, and 140 DAF (days after flowering of female flower) of 10 years “XC” tree by RNA-seq. 21 MADS-box genes were expressed in at least one stage of fruit development (Figure 9A and Supplementary Table 10). Ten genes (*DlMADS75*, *70*, *59*, *55*, *72*, *18*, *114*, *29*, *49*, *68*) were only expressed at low levels in the first two stages of fruit development (DAF 80 and DAF 100). *DlAP3-1*, *DlAP3-2*, and *DlMADS71* were down-regulated gradually during developmental stages and *DlFLC* showed the reverse trend. *DlSTK* and *DlSHP* exhibited the highest expression levels in DAF 80, while the expression of *DlSTK* rose in later stages and *DlSHP* decreased until it was not expressed at all. Increasing expression from DAF 80 to DAF 130 was found in *DlSEP1* and *DlSEP2* indicating that they may be involved in the fruit ripening process. It is significant that *DlMADS53* (*M*α) showed an extremely high expression level suggesting important functions in longan fruit development. The RNA-seq results of *DlAP3-2*, *DlSTK*, *DlFLC*, *DlFUL*, and *DlMADS53* were corroborated by qRT-PCR. The relative expression levels were in agreement with the FPKM, confirming the accuracy of our transcriptomic analysis (*R*^2^ > 0.9; Figure 8B).

## Discussion

Given the developments in genome sequencing, there are numerous reports on the important MADS-box family in various plants such as Arabidopsis (Parenicova et al., 2003), rice (Arora et al., 2007), soybean (Fan et al., 2013), and banana (Liu et al., 2017). In this study, we completed the identification of the MADS-box gene in “SX” longan genome for the first time, and 114 MADS-box members were identified and classified into type I: *M*α (39), *M*β (10), *M*γ (14), and type II: *MIKC^C^* (36), MIKC* (14) (Supplementary Table 2). Combined with the relative representative plant species, it is possible to understand the evolution of MADS-box genes.

### Evolution of MADS-Box Family

In this study, we identified the MADS-box genes from 15 representative species (Figure 1). The primitive lower plant, *Chlamydomonas reinhardtii*, only contained two genes belonging to both type I and type II, in accordance with the view that these two types of genes appeared by a duplication event before the divergence of plants and animals (Alvarez-Buylla et al., 2000). Overall, the number of MADS-box genes in Angiosperms is larger than that in Pteridophyta, Bryophyta, and Alga. A large expansion of *MIKC^C^* genes was found in basal angiosperms, given that flowering is a typical feature of high plants, we predict *MIKC^C^* genes may be endowed with new functions as plants evolved especially in flowering and development. In monocots and eudicots, type II genes showed relative conservation and the number of type I genes varied greatly in eudicots. It was found that longan contained the largest number of family genes, and we speculate that members of the MADS-box family were likely more active for tandem duplication after the γ-WGD event in longan than other eudicot species (Table 1). Furthermore, the degree of retention of type I genes seems to be higher than in other angiosperms. MIKC* genes expanded much more and they mainly originated from WGD/Segmental and dispersed duplication.

In Sapindaceae, the three examined genomes have no additional WGD after the γ-WGD, while, both longan and lichee have large numbers of MADS-box genes as the tandem duplication events frequently occurred in the longan genome. This phenomenon indicated that longan and lichee probably have complex molecular mechanisms for flower development due to the interaction of large sets of MADS-box genes for the regulation of gene networks. Sapindaceae could be classified into two subfamilies including Sapindoideae and Dodonaeoideae (Harrington et al., 2005). The divergence of the two subfamilies resulted in a great variation in the number of the type I genes particularly for *M*β and *M*γ (Figure 1), which was probably caused by the functional redundancy of the two subgroups of MADS-box genes since these genes have few reports detailing their functions (Masiero et al., 2011). *MIKC^C^* genes were demonstrated to be involved in floral organ identity, the control of flowering time, and seed development. The relatively consistent number of *MIKC^C^* genes in Sapindaceae is likely attributed to the functional conservation of these genes in floral organ development.

### *Dof* Family May Regulate *MIKC^C^* Genes in the Flowering Process

The potential transcription factors which control *MIKC^C^* genes were identified, and 26 TF binding sites were found in promoters of most *MIKC^C^* genes (Supplementary Table 7). The majority of TFs contain *C2H2* zinc finger domain such as the *Dof* family, which is involved in photoperiod inducement of flowering and flowering development (Yanagisawa, 2002). In Arabidopsis, *CYCLING DOF FACTOR* (*CDF*) is the well-known inhibitory factor of flowering which can repress the expression of *FT* and *CONSTANS* (*CO*), it can be degraded by ubiquitin-protein *KELCH REPEAT, F-BOX 1* (*FKF1*) (Imaizumi et al., 2005). *OBF4 Binding Protein 3* (*OBP3*) and *Cogwheel 1* (*COG1*) modulate phytochrome to further influence flowering (Park et al., 2003; Ward et al., 2005). Other research showed that maize transcription factor *Zmdof1* regulates pollen formation and development (Chen et al., 2012). In rice, overexpressing *OsDof12* led to early flowering and up regulation of *OsMADS14* (Li et al., 2009). The previous study has demonstrated that *Dof* proteins were able to recognize (A/T)AAAG sequence as the core motif (Chen et al., 1996; Yanagisawa and Schmidt, 1999). In this study, similar binding sites of *CDF5*, *COG1*, *FOXP1*, *OBP3*, and other *Dof* genes were found in cis-motif 2, 3, 4, and 5 (Figure 5B). It could be speculated that *Dof* TFs may directly regulate *MIKC^C^* genes in longan, however, this speculation needs further investigation.

*BBR-BASIC PENTACYSTEINE* (*BPC*) identified in Arabidopsis is a small transcription factor family of seven members (Meister et al., 2004). *BPC1* binds to the *STK* promoter at GA consensus sequences and regulates the expression of *STK* (Kooiker et al., 2005). In addition, chromatin immunoprecipitation analysis showed that *BPCs* also bind to the GA boxes *in vivo*, and their mutation can result in suppressed expression of the *STK* promoter (Simonini et al., 2012). Our data showed that three genes of the *BBR-BPC* family probably bind sequences in cis-motif 1, which provides evidence that *BPC* binding sites are important for *STK* and other *MIKC^C^* genes (Figure 5B). Moreover, *AGAMOUS-LIKE 42* (*AGL42*) is a member of the *SOC1* subfamily and controls the floral transition in the axillary meristem, and *SOC1* directly binds to CArG-boxes from other *SOC1* class genes (Dorca-Fornell et al., 2011). *SOC1* plays a central role in the flowering pathway (Moon et al., 2003; Yoo et al., 2005), and a recent study showed that *SOC1* also binds to its own locus and several flowering genes including the MADS-box members (Immink et al., 2012). Cis-motif 6 annotated as *SOC1* binding sites was found in the promoter of 15 *MIKC^C^* genes (Supplementary Table 7), perhaps *SOC1* class members regulate other MADS-box genes during the longan flowering process.

### Functional Conservation of ABCDE Model Genes in Longan

Longan is monecious and very little is known on longan floral biology. We collected four whorls of organs and ovaries from male and female flowers to predict the ABCDE model of longan (Figure 6). The expression of A-class *DlAP1* was notably higher in sepal than in other floral organs indicating the sepal identity of A function. Its close paralogs *DlFUL* was mainly expressed in the ovary. In Arabidopsis, *FUL* is required for valve differentiation and expansion in the ovary after fertilization (Zhang Y. et al., 2016). Generally, B class genes were restricted to expressed in the second and third whorls in model plants (Jack et al., 1992; Guo et al., 2015). In our results, *DlPI* was only detected in petals and stamens, which is in line with its expected function in petal and stamen identity specification. *DlAP3-1* was also expressed in the other two organs (sepal and carpel), indicating neo-functionalization in longan after the divergence of *PI* and *AP3*. Regarding the C class, *AG* was the first floral organs identity gene found to determine the stamen and carpel in Arabidopsis (Yanofsky et al., 1990). *DlAG* displayed a higher expression level in the stamen and carpel than the other organs, suggesting that *DlAG* maintains the C function of specifying both male and female reproductive organs in longan. A recent study revealed that Arabidopsis *AG* regulated sepal senescence (Jibran et al., 2017). In this study, the transcripts of *DlAG* were also found in sepals, supporting a potential role for sepal senescence in longan. Both *DlSHP* and *DlSTK* were relatively highly expressed in the ovary, we, therefore, hypothesized that *DlSHP* and *DlSTK* perform class D functions in longan ovule development.

All four members (*SEP1/2/3/4*) play redundant functions in determining floral organ identity in Arabidopsis (Zahn et al., 2006). A similar result was found in longan with four *DlSEP* having different expression profiles in tested organs. *DlSEP1* and 3 seem to account for a major position of E function due to their wider and higher expression level. *DlSEP4* might regulate the ovule development together with *DlFUL*, *DlSHP*, and *DlSTK* in the early stage. *AGL6* class was closely related to *SEP* in the phylogeny (Figure 2; Becker and Theissen, 2003), and orthologs of *AGL6* in rice, maize, and petunia have been regarded as regulators for multiple floral organ identity like Class E function (Ohmori et al., 2009; Rijpkema et al., 2009). Our analysis showed that *DlAGL6* possibly controls sepal, petal, and stamen of longan, not like *TaAGL6* which is required for all four whorls of wheat floral organs (Kong et al., 2021). Overall, the expression patterns of ABCDE model genes in longan are similar to typical models in Eudicots (Figure 6D).

### The MADS Genes Are Involved in Flowering and Respond to Potassium Chlorate

MADS-box genes play significant roles in flowering processes in plants. Expression analysis allowed us to predict the function of longan MADS-box genes. Our results revealed a diverse expression pattern in different longan tissues. Compared to previous studies (Kofuji et al., 2003), the expression of most genes in type I and MIKC* was not detected, with their function remaining unclear and it is possible they are only be expressed in specific cells or under specific conditions. Nevertheless, we found several genes such as *DlMADS21* and *DlMADS53* in root and pulp, suggesting these subfamilies with few studies never play a negligible role (Supplementary Figure 5). These type I and MIKC* genes should be investigated in future work.

Flowering is the key factor in yield determination (Bangerth, 2009), but the problem of poor production persisted for a long time owing to low flowering rates in longan. Normally, flower induction in longan needs a period of low temperature (Suttitanawat et al., 2012), and longan can respond to KClO_3_ for floral bud formation. The molecular mechanism associated with off-season flowering induction is of great value in scientific research and is of practical significance.

*MIKC* genes are of vital importance in reproductive development, especially the well know ABCDE model (Coen and Meyerowitz, 1991). In the present study, many *MIKC* genes seem to involve in the longan flowering process, as highly expressed genes usually play important roles in plant development. For example, *DlAP1*, *DlPI*, *DlAGL6* were highly expressed specifically in flower buds and flowers (Figure 7), which highlights their key roles in flowering. *DlAG, DlSHP, DlFUL, DlSEP1/2/3* showed high expression levels in multiple tissues, inferring their function in regions beyond just flowers. More importantly, 11 *DlMADS* were up-regulated after KClO_3_ treatment (Figure 8A), highlighting the involvement of MADS-box genes in off-season FI of longan.

Normally, *LFY* was considered as a determinant of flower initiation that plays a central role in flower pathways (Fornara et al., 2010). Recently, *LFY* was confirmed as a pioneer transcription factor to promote floral transition via upregulating *AP1* (Jin et al., 2021). However, the ortholog of *LFY* in longan showed no apparent changes during flower induction in “SJ” (Jue et al., 2019). On further investigation, we found the expression of *DlAP1* in “SJ” was significantly lower than in “SX” especially in the floral primordia stage, even though it was induced during floral transition in both accessions (Supplementary Figure 8). This result implied that the molecular pathways of flowering in “SJ” may differ from those in the other longan accessions. One A-class gene, *DlMADS98*, showed a higher expression level in “SJ.” It is likely that the *DlMADS98* displayed function complementation with *DlAP1* in off-season FI.

In Arabidopsis, *FLC* and its five homologs, *MAF1-5* co-regulates flowering time by temperature-dependent alternative splicing (Scortecci et al., 2001; Bastow et al., 2004). Flowering induction in longan also requires low temperature (vernalization), thus, *FLC* might be functionally constrained in longan. *DlFLC* showed extensive-expression especially in longan vegetative and reproductive tissues and slightly decreased during off-season flowering. Significantly, the expression of *DlFLC* was lower in “SJ” than that in “SX” during the whole stages (Supplementary Figure 8). Given that *FLC* is regarded as a repressor in different flowering pathways (Rouse et al., 2002), we could speculate that *DlFLC* may be an inhibitor of off-season FI in longan. *SVP* is another important gene in response to environmental temperature to regulate plant flowering. The *SVP* class showed obvious expansion and the collinearity analysis implied it experienced tandem gene duplications like the *DAM* genes in Rosaceae (Supplementary Figure 3). RNA-seq and qRT-PCR analysis showed that *SVP* class genes such as *DlMADS82* tend to be expressed in leaf tissue and dormant buds, and this gene was also suppressed by KClO_3_ (Figures 7, 8). This leads us to speculate that *SVP* class genes could regulate vegetative development and be responsible for apical bud formation in response to dormancy.

Moreover, *SOC1* responds to multiple floral induction pathways including photoperiod, vernalization, and autonomous (Lee et al., 2000). It acts downstream of *FLOWERING LOCUS T (FT)* and regulates the expression of *LFY* which promotes transcription of *AP1* (Immink et al., 2012). On the other hand, it was also identified as a *FLC* suppressor (Lee et al., 2000). Contrary to the previous study, *DlSOC1* exhibited little expression in flowers or floral buds and was down-regulated after KClO_3_ treatment. It is also notable that *DlMADS111* (*SOC1* clade) and *DlSOC1* showed lower expression levels in “SJ.” Whether *SOC1* clade genes have altered functions in longan remains to be elucidated. From these results, it seems that genes in *DlFLC*, *DlSVP*, and *DlSOC1* have similar functions in maintaining vegetative growth and inhibiting floral transition, with further studies being required to verify the function of these genes.

### Potential Roles of Longan MADS Genes in Fruit Development

Longan fruit, which contains many nutrients such as vitamins, carbohydrates, mineral elements, and amino acids, plays an important role in human anti-cancer, anti-aging, and brain development (Rangkadilok et al., 2005). The growth and development of longan fruit usually take 100–150 days. During this period, there are obvious changes in fruit size, nutritional composition, sweetness, and flavor, which are regulated by external environmental conditions, plant hormones, and many genes. In the present study, 9 of the MADS-box genes were highly expressed in fruit (Figure 9). *FRUITFULL FUL* is known for its role in controlling flowering time, carpel identity, fruit development, and leaf morphology (Coen and Meyerowitz, 1991; Gu et al., 1998; Torti et al., 2012). Functional studies in multiple species indicated that *FUL* orthologs have a conserved function for regulating fruit development even in fruits with diverse morphologies and structures (Jaakola et al., 2010; Bemer et al., 2012; Pabón-Mora et al., 2012; Fujisawa et al., 2014). Not surprisingly, in longan, *DlFUL* showed extremely high expression levels in young fruit, pericarp, and flowers, suggesting that this gene also has a conserved function for regulating the flowering time and fruit development (Figures 7, 9).

Recent research showed that ectopic expression of *MdPI* controls apple fruit tissue growth and shape, indicating that the functions of B class genes are not limited to the development of petals and stamen (Yao et al., 2018). Analysis of *Sl-AGL11* overexpression in tomatoes revealed that the D class gene could regulate early fleshy fruit development (Huang et al., 2017). In this study, two *DlAP3* genes were not only expressed in flowers, but also expressed in fruit (seed, pericarp, and pulp) with different patterns during fruit development. The transcript of *DlSTK* has detected all the examined fruit organs with high levels in the pulp. It is likely that longan B and D class genes experienced different evolutionary fates from Arabidopsis and played functions for fruit development. In addition, a preview study has reported that *LeMADS-RIN* is necessary for fruit ripening at the tomato ripening-inhibitor (rin) locus (Vrebalov et al., 2002). Arabidopsis *SEP* genes were the homologs of *LeMADS-RIN*. We determined that *DlSEP1* and *2* were extremely highly expressed with expression tending to increase during fruit development, which suggests *DlSEP1* and *2* may act as regulators of longan fruit ripening, with further studies being required to verify their function. It is noteworthy that a type I gene *DlMADS53* only expressed in the pulp of longan and remained at high expression levels during fruit ripening. To the best of our knowledge, there is no study about the orthologs of *DlMADS53* in plants. This result provides a new clue for the functional study of the MADS-box in the plants.

## Conclusion

This study identified 114 MADS-box genes including 63 types I and 51 types II genes in the longan genome. Thirteen subfamilies of *MIKC* genes were identified through phylogenetic analysis and the main difference between *MIKC^C^* and MIKC* proteins were the K-box domain. Analysis of cis-elements revealed that *Dof* transcription factors might directly regulate the *MIKC^C^* genes. Phylogeny and gene expressional analysis showed that the composition and expression of the ABCDE genes were conserved in longan. We also provided expression information for *DlMADS* in vegetative and reproductive tissues and this data led us to conclude that *MIKC^C^* genes play crucial roles in flowering. Several genes such as *DlSTK*, *DlSEP1/2*, and *DlMADS53* could be involved in fruit growth and ripening. In summary, this comprehensive analysis provided the basic resources to examine the molecular regulation of MADS-box genes in the longan reproduction process.

## Data Availability Statement

The datasets presented in this study can be found in online repositories. The names of the repository/repositories and accession number(s) can be found below: https://www.ncbi.nlm.nih.gov/, PRJNA741049.

## Author Contributions

JZ conceived the study and designed the experiments. BW, WH, YF, XF, JF, TZ, SZ, RM, and JZ carried out the experiments and analyzed the data. BW and JZ wrote the manuscript. All authors read and approved the final manuscript.

## Conflict of Interest

The authors declare that the research was conducted in the absence of any commercial or financial relationships that could be construed as a potential conflict of interest.

## Publisher’s Note

All claims expressed in this article are solely those of the authors and do not necessarily represent those of their affiliated organizations, or those of the publisher, the editors and the reviewers. Any product that may be evaluated in this article, or claim that may be made by its manufacturer, is not guaranteed or endorsed by the publisher.

## Footnotes

1
http://www.arabidopsis.org/

2
https://phytozome.jgi.doe.gov/pz/portal.html

3
http://gigadb.org/

4
https://www.ncbi.nlm.nih.gov/Taxonomy/CommonTree/wwwcmt.cgi

5
http://Pfam.sanger.ac.uk/

6
https://web.expasy.org/protparam/

7
http://gsds.gao-lab.org/

8
http://meme-suite.org/tools/meme

9
https://www.ebi.ac.uk/interpro/search/sequence/

10
http://bioinformatics.psb.ugent.be/webtools/plantcare/html/

11
http://jaspar.genereg.net

12
https://meme-suite.org/meme/tools/tomtom

13
https://sg.idtdna.com/pages/tools/primerquest

14
http://www.phytosystems.ulg.ac.be/florid/
