# Supplementary material for: Comparative Analysis of the MADS-Box Genes Revealed Their Potential Functions for Flower and Fruit Development in Longan (Dimocarpus longan)
Source: Front Plant Sci. 2022 Jan 27;12:813798. doi: 10.3389/fpls.2021.813798 (PMC8829350; doi:10.3389/fpls.2021.813798)
Supplement: Supplementary file 1 [file Data_Sheet_1.docx]

Supplementary Material

## Supplementary Figures

**
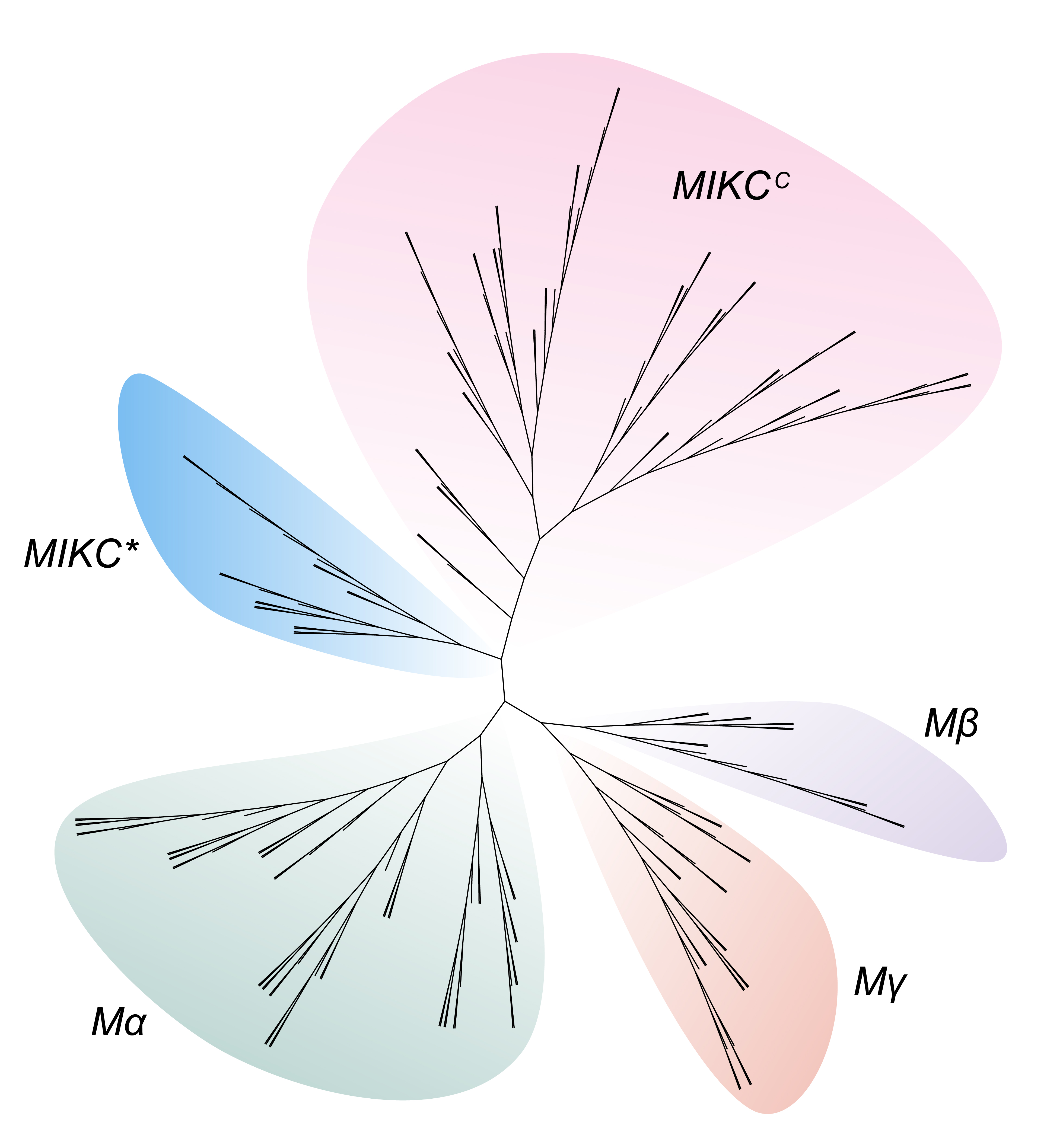
**

**Supplementary Figure 1.** Phylogenetic trees of longan and Arabidopsis MADS-box proteins. The Neighbor-joining (NJ) tree was drawn in MEGA X with 1000 bootstrap. The green, purple, croci, bule and red areas indicate *Mα*, *Mβ*, *Mγ*, *MIKC** and *MIKC^C^* groups.





**Supplementary Figure 2.** Phylogenetic tree of *MIKC^C^* genes built with Arabidopsis, longan (*Dl*), litchi (*LITCHI*) and yellowhorn (*EVM*) protein sequences by neighbor-joining method.


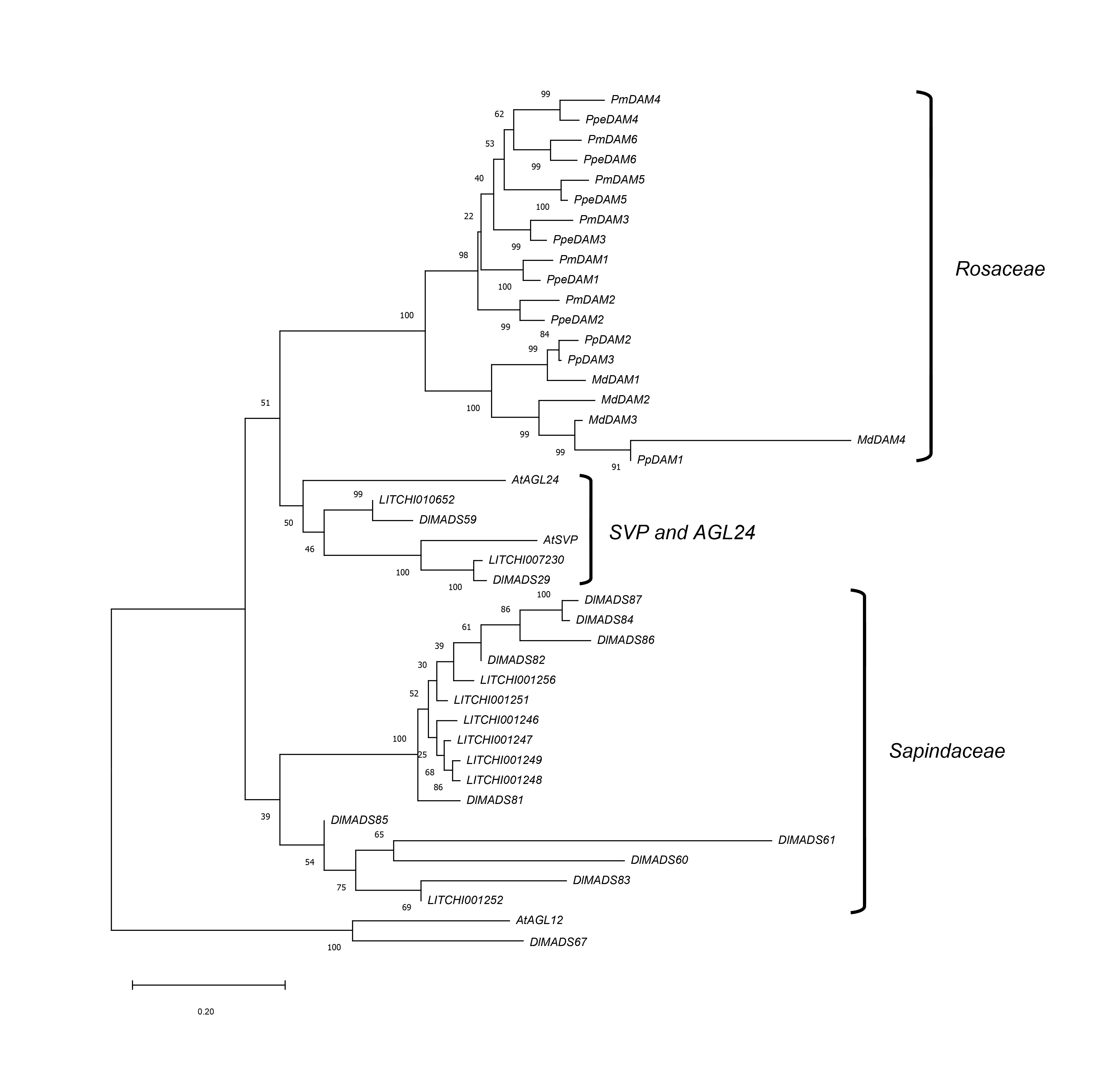


**Supplementary Figure 3.** Phylogenetic trees of *SVP* class genes of longan (*Dl*) and Arabidopsis (*At*), peach (*Ppe*), plum (*Pm*), pear (*Pp*) and apple (*Md*). *AtAGL12* and *DlMADS67* were used as outgroups.


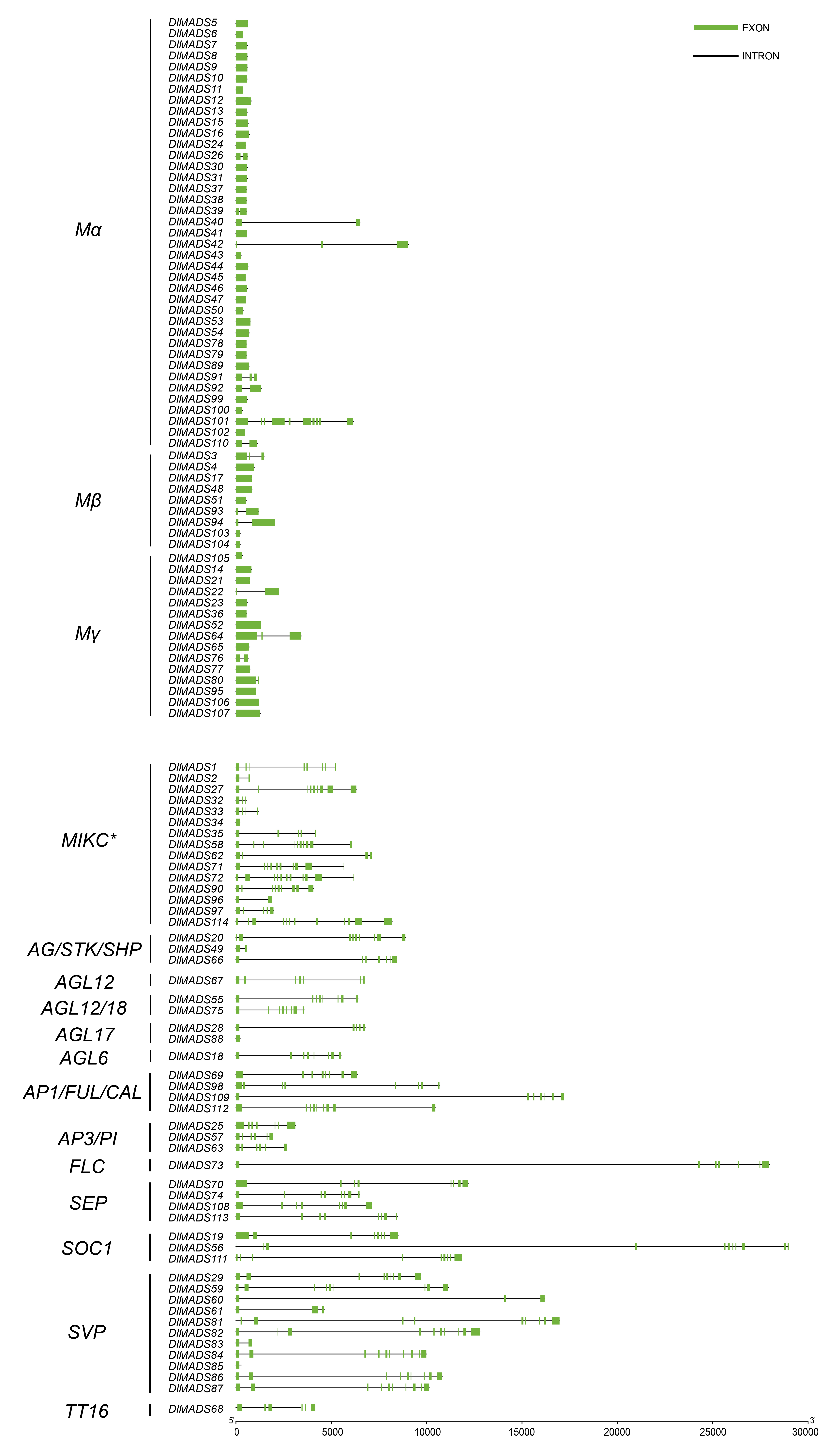


**Supplementary Figure 4.** Gene structure analysis of MADS-box genes in longan. Green solid boxes represent exons and black lines represent introns.


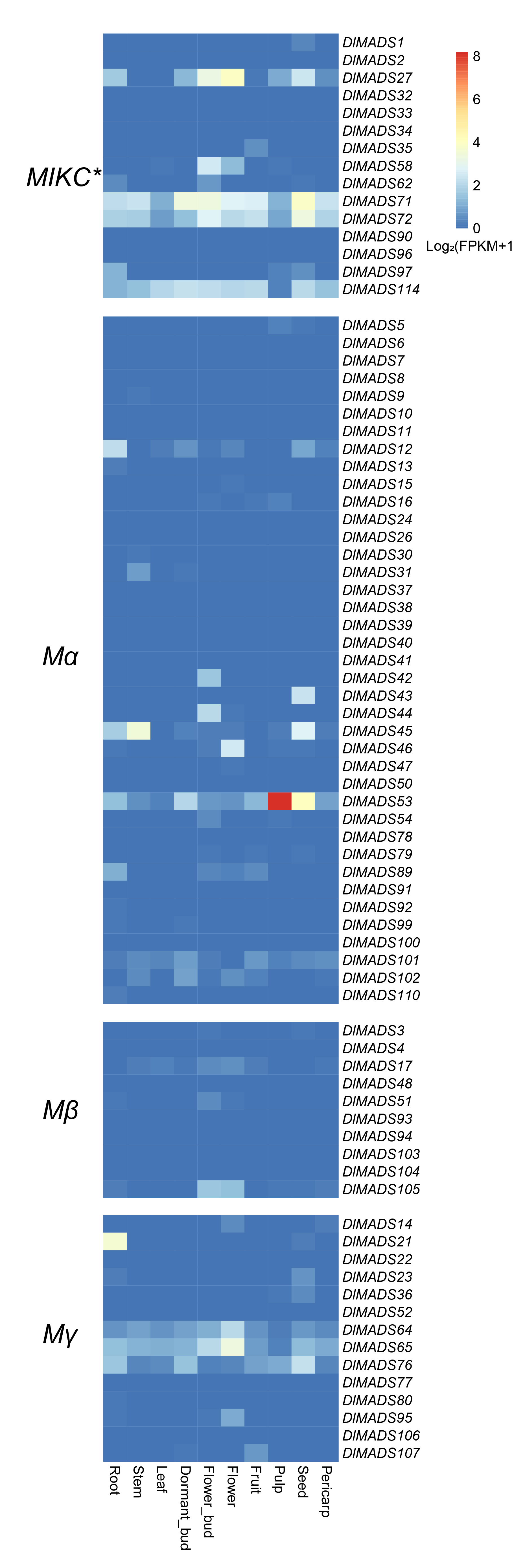


**Supplementary Figure 5.** Expression heat maps of type I and *MIKC** genes in multiple longan tissues.


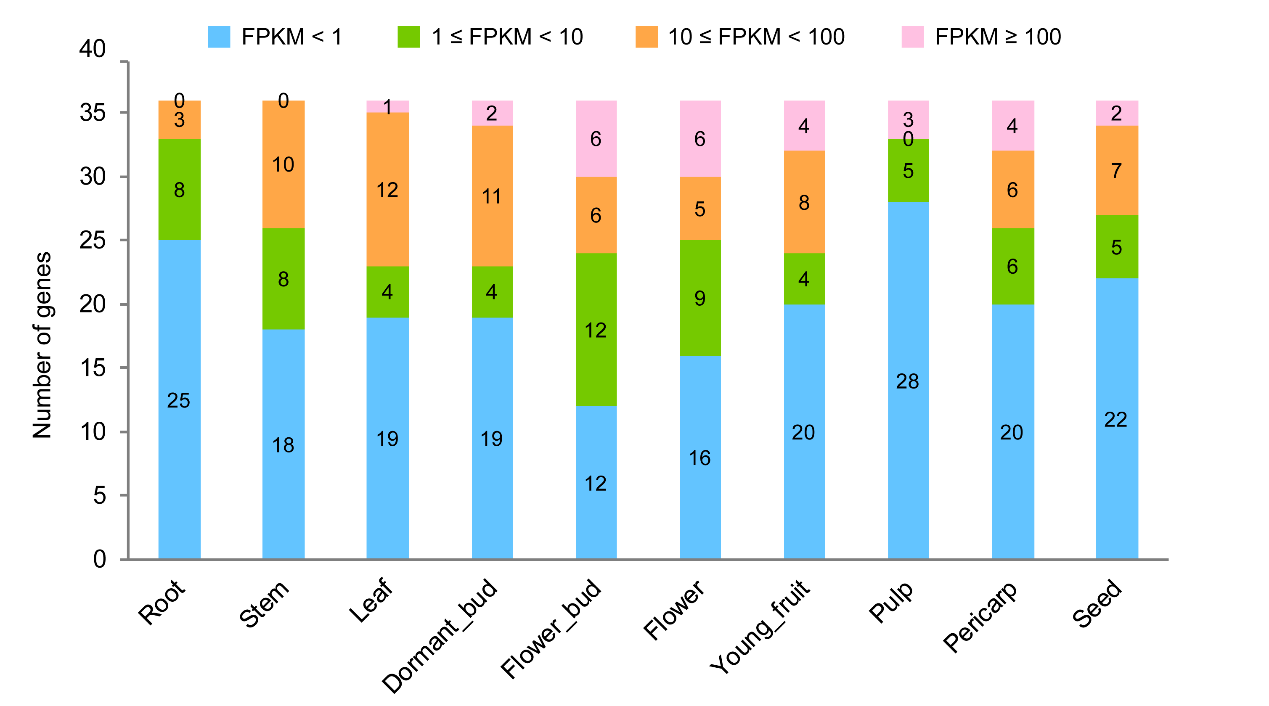


**Supplementary Figure 6.** Number of *MIKC^C^* genes expressed in root, stem, leaf, dormant bud, flower bud, flower, young fruit, pulp, pericarp and seed.


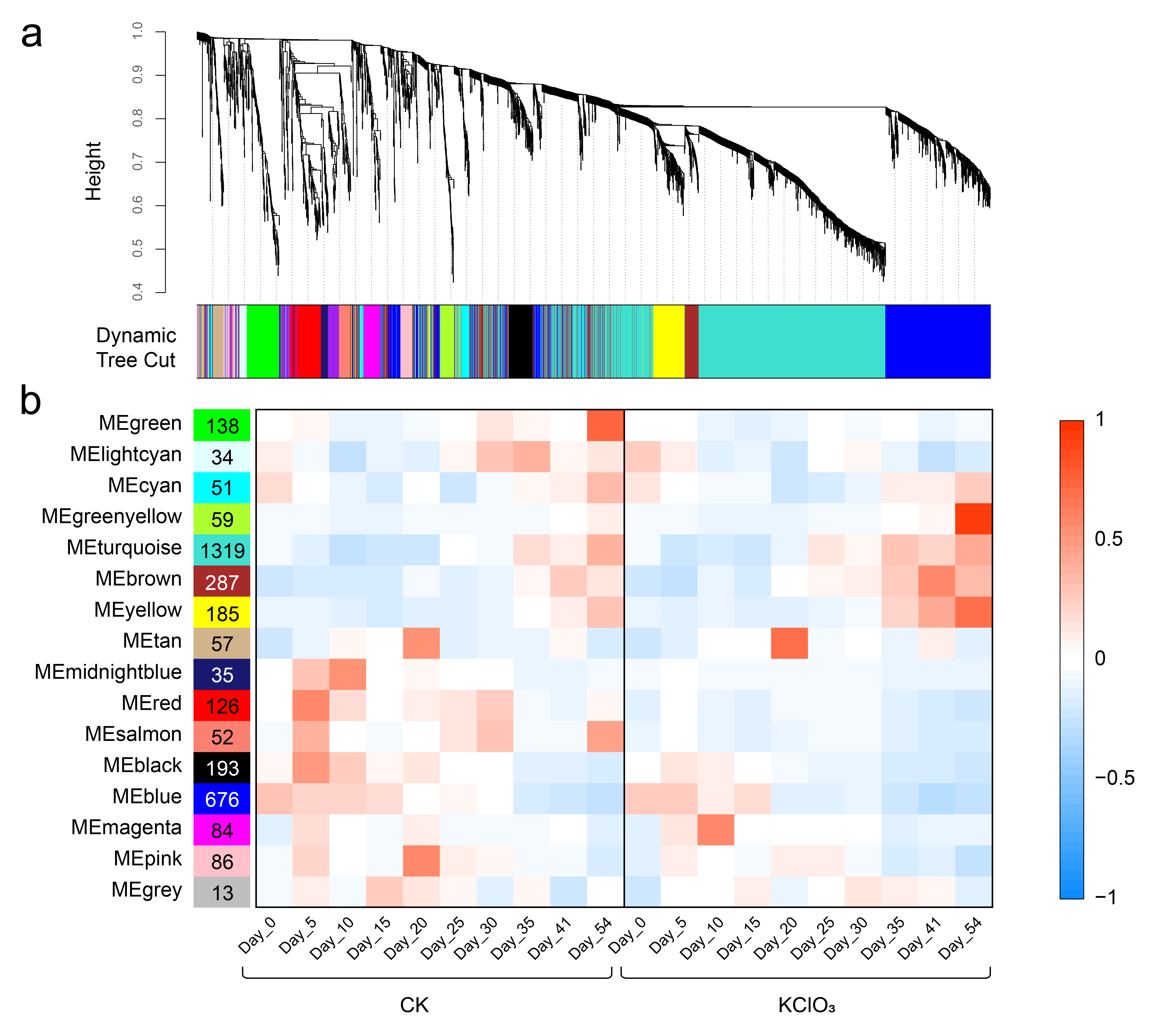


**Supplementary Figure 7. Weighted Gene Co-Expression Network Analysis (WGCNA) of genes in off-season flower induction. a.** Hierarchical cluster tree showing co-expression modules identified by WGCNA. **b.** The module-trait relationships show the correlation between gene expression level and trait intensity.

**
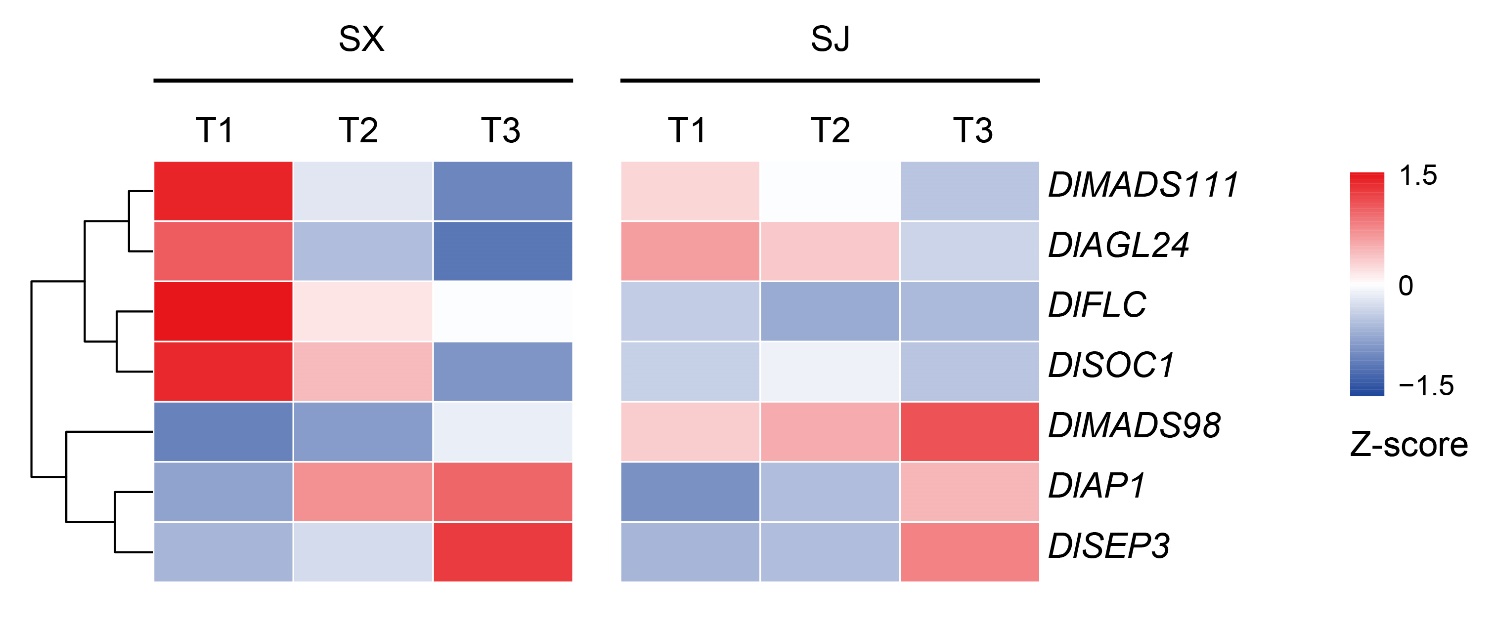
**

**Supplementary Figure 8. Heatmaps of differentially expressed MADS-box genes between ‘SX’ and ‘SJ’ longan.** T1: dormant bud; T2: floral primordia; T3: floral organ formation. FPKM data for gene expression levels were normalized by the Z-score.
